# A Novel AI-Guided Hormone Replacement System Engineered to Maintain Physiologic Levels of 17β Estradiol and Progesterone

**DOI:** 10.3390/jpm16100514

**Published:** 2026-09-30

**Authors:** Fatima Al-Reshawee, Hussein Alburkat

**Affiliations:** 1 Department of Medical Microbiology, College of Medicine, Al-Muthanna University, Samawah 66001, Iraq; hussein.alburkat@mu.edu.iq; 2 Department of Virology, Faculty of Medicine, Helsinki University, 00014 Helsinki, Finland

**Keywords:** HRT, CVD, estrogen, progesterone, AI model

## Abstract

Some cases of primary and secondary amenorrhea are associated with significant symptoms and an increased risk of multiple diseases, including cardiovascular disease (CVD), osteoporosis, genitourinary dysfunction (urinary urgency and incontinence), vasomotor symptoms such as hot flashes and night sweats, and central nervous system manifestations resulting from estrogen (17-Beta estradiol) deficiency. However, current hormone replacement therapy (HRT) has important limitations in reproducing the physiological actions of endogenous 17-Beta estradiol due to the route of administration, dosing patterns, and adverse effects of synthetic hormones. This paper proposes a novel therapeutic system designed to overcome these limitations. The system consists of two main components: (1) the production of human-identical 17β-estradiol and progesterone using genetically engineered yeast, and (2) the delivery of these hormones via a subcutaneous pump programmed to maintain physiologic pulsatile secretion. An AI model assists in determining the optimal hormone dose and timing for each patient based on individualized parameters such as body weight, SHBG levels, and overall health status.

## 1. Introduction

During the reproductive years, women naturally secrete 17β-estradiol and progesterone in a regular and balanced manner that provides strong effects on multiple systems [1,2]. This physiologic balance allows each hormone to exert its beneficial effects without the adverse consequences of unopposed activity. Both 17β-estradiol and progesterone exert significant effects on multiple organ systems, such as the central nervous system, where 17β-estradiol plays a neuroprotective role by promoting neuronal survival, enhancing synaptic plasticity, and supporting cognitive functions such as memory and learning; it also modulates neurotransmitter systems and reduces age-related neurodegenerative changes. Meanwhile, progesterone contributes to neural protection through anti-inflammatory mechanisms, regulation of apoptotic pathways, and modulation of neurotrophins, including brain-derived neurotrophic factor (BDNF), which supports neuronal resilience and synaptic integrity. Together, 17β-estradiol and progesterone interact with specific receptors and intracellular signaling pathways to maintain structural and functional brain homeostasis [3,4,5]. Beyond their role in the central nervous system, 17β-estradiol and progesterone exert systemic effects that extend to the cardiovascular system. 17β-estradiol exerts multiple cardioprotective actions, including raising high-density lipoprotein (HDL) cholesterol and lowering low-density lipoprotein (LDL) cholesterol, improving insulin sensitivity, and promoting vasodilation through enhanced release of nitric oxide (NO). 17β-estradiol also protects against thrombosis by stimulating the production of NO and prostacyclin (PGI_2_) [6,7,8]. Progesterone complements 17β-estradiol by balancing its effects on fat deposition: its role in pancreatic *β*-cell function and insulin secretion remains an area of active investigation with mixed findings across studies [9]. Similarly, hormonal balance is essential for skeletal integrity, 17β-estradiol plays an important role in maintaining bone homeostasis by regulating the balance between bone resorption and bone formation. It suppresses osteoclast activity, promotes osteoblast survival, and modulates key molecular pathways such as the RANKL/OPG system, thereby preventing excessive bone loss. Following menopause, 17β-estradiol deficiency leads to increased bone resorption, reduced bone mineral density (BMD), and a higher risk of osteoporosis and fractures [10,11]. Progesterone also contributes to skeletal health by stimulating osteoblast differentiation and supporting bone formation. Evidence suggests that combined 17β-estradiol–progestin therapy results in greater improvements in bone mineral density compared to 17β-estradiol alone, particularly in the lumbar spine [11]. In the genitourinary tract, 17β-estradiol and progesterone play essential roles in the integrity and function of the genitourinary system. 17β-estradiol receptors are widely distributed across the lower urinary tract and genital tissues, where hormonal stimulation maintains tissue structure, mucosal health, and local immune defense. Menopausal decline in 17β-estradiol disrupts the urinary microbiome, reducing protective *Lactobacillus* species and contributing to symptoms such as urinary urgency, incontinence, and bladder pain [12].

At the level of thermoregulation, 17β-estradiol plays a central role in thermoregulation by stabilizing hypothalamic control of body temperature. Declining 17β-estradiol levels during menopause or primary and secondary amenorrhea narrow the thermoneutral zone, leading to inappropriate heat dissipation responses manifested as hot flashes and night sweats. Emerging evidence highlights the role of hypothalamic KNDy neurons and neurokinin B signaling in the pathophysiology of vasomotor symptoms [13]. Hormone therapy, particularly systemic 17β-estradiol, remains the most effective intervention for moderate to severe vasomotor symptoms. Progesterone may additionally contribute to sleep stabilization and nocturnal symptom improvement [14].

Despite the wide physiological benefits of 17β-estradiol and progesterone, significant challenges remain in using hormone replacement therapy (HRT) as a substitute for endogenous hormones, particularly in women with primary ovarian insufficiency who require long-term HRT use. These challenges are largely related to therapy-associated adverse effects. These limitations underscore the need for a next-generation hormonal replacement system that addresses the challenges of timing, dosing, methods of administration, and the chemical form of synthetic hormones, while more closely maintaining the physiologic pattern of hormone release.

Current hormone replacement therapy (HRT) has limitations in fully replicating the physiological effects of endogenous hormones [15]. Oral 17β-estradiol (E2) therapy is associated with an increased risk of venous thromboembolism, pulmonary embolism, and ischemic stroke, largely due to hepatic first pass metabolism and increased synthesis of coagulation factors, with risk being particularly elevated in older women or when therapy is initiated late after menopause [1,16,17,18]. The initiation of HRT late after menopause may be associated with an increased risk of coronary events, potentially related to pre-existing atherosclerotic plaque instability [19]. Long-term combined oral 17β-estradiol–progestin therapy has also been linked to an increased risk of breast cancer [20], while unopposed 17β-estradiol-based therapy increases the risk of endometrial hyperplasia and carcinoma [21]. Furthermore, 17β-estradiol-based therapy has been linked to an increased incidence of gallstones and cholecystitis [22].

These limitations are influenced by several key factors. First, the route of administration plays a critical role. Oral HRT undergoes first pass hepatic metabolism, leading to increased hepatic protein synthesis and altered coagulation profiles [16,17]. Second, the dosing pattern, current delivery methods cannot replicate the accurate, pulsatile control of hormone quantities within a given time frame [23]. For example, transdermal therapy provided relatively static hormone exposure, which may not account for inter-individual variability in hormone metabolism and response, and sustained receptor stimulation, rather than dynamic physiologic fluctuations. Third, the chemical composition of some synthetic hormones, particularly certain progestins, may result in off-target receptor binding, reducing physiological specificity compared to endogenous hormones [24]. Together, these limitations highlight the need for more adaptive and individualized hormone replacement strategies that aim to maintain physiologic hormone levels within safe and clinically relevant ranges.

## 2. Hypothesis

The aim of the proposed system is to overcome the limitations of current hormone replacement therapy (HRT) and to restore the physiological benefits of 17β-estradiol and progesterone. This hypothesis is primarily developed for women with primary ovarian insufficiency (POI), a population characterized by long-term hormone deficiency, an extended treatment horizon often spanning several decades, and a correspondingly strong rationale for physiologic, cycle-matched hormone replacement. The production and delivery architecture proposed here is not inherently specific to POI and may be extensible to other hormone-deficient states, including surgical and natural menopause; however, these populations differ in treatment duration, baseline age-related risk, and target hormone profile (a stable physiologic range rather than a cycling pattern), and would require population-specific calibration and independent validation. Hypothalamic amenorrhea is excluded from the scope of this hypothesis, as it reflects a functionally distinct mechanism—intact ovaries suppressed by hypothalamic–pituitary dysfunction, for which addressing the underlying cause is often the preferred first line approach rather than long-term exogenous hormone replacement.

### 2.1. Human-Identical Hormone Production

**Figure 1:**
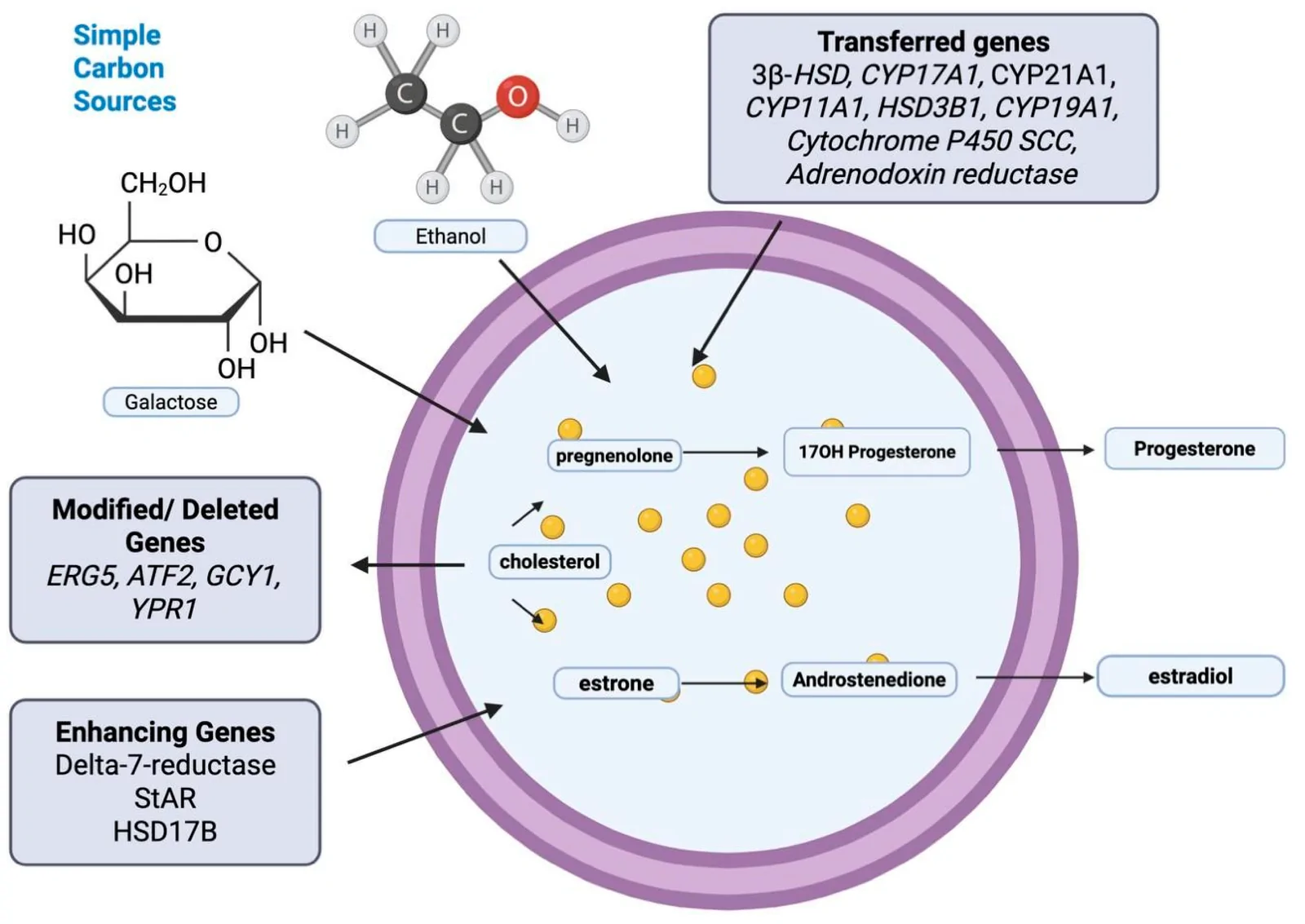
The metabolic engineering of a microorganism for the production of 17β-oestradiol and progesterone. A schematic representation of the engineered metabolic pathway for the biosynthesis of steroid hormones from simple carbon sources in a host cell (represented by the purple circle). Galactose (a hexose sugar) and ethanol are utilized as the primary simple carbon sources for metabolism. Cholesterol serves as the initial steroid precursor, which is subsequently converted through various steps to produce progesterone and estrogen. Designed by Biorender.com.

Some current hormone replacement therapies utilize synthetic or non-human-derived steroid compounds whose molecular structures differ from endogenous human hormones. These structural modifications include additional functional groups, altered stereochemistry, or substitution patterns that influence receptor binding affinity, metabolic pathways, and biological activity. For example, synthetic progestins such as medroxyprogesterone acetate possess structural alterations that enable interaction with non-progesterone steroid receptors, including glucocorticoid and androgen receptors, potentially producing off-target physiological effects [24]. Conjugated equine estrogens (CEEs) are a complex mixture of estrogenic compounds derived from the urine of pregnant mares. Many components (e.g., equilin and equilenin) are not present in humans [25]. These molecules differ structurally from human 17β-estradiol, resulting in distinct receptor binding patterns, metabolism, and biological effects. The non-human origin and heterogeneous composition make CEEs pharmacologically different from endogenous 17β-estradiol. Despite the availability of bioidentical hormones represented by micronized progestins and micronized 17β-estradiol, their production is primarily based on semi-synthetic chemical processes starting from plant-derived phytosterols converted into C19 steroids (such as 9-OHAD, ADD, and androstenedione). Microbial biotransformation may be used in early steps to convert sterols into key intermediates such as androstenedione after modifying major and minor metabolic pathways [26]; however, the final hormone products, including estradiol and progesterone, are typically obtained through subsequent chemical synthesis rather than direct microbial biosynthesis. In contrast, the proposed system utilizes a de novo biosynthesis approach using yeasts such as *Saccharomyces cerevisiae*. In this strategy, 17β-estradiol and progesterone are synthesized directly from simple carbon sources such as ethanol and galactose after transferring key genes from humans and mammals (e.g., 3β-HSD, *CYP17A1*, *CYP21A1*, *CYP11A1*, *HSD3B1*, *CYP19A1*, cytochrome P450 SCC, and adrenodoxin reductase), while modifying or deleting others (*ERG5*, *ATF2*, *GCY1*, *YPR1*) to eliminate unwanted interactions during pregnenolone reactions (Figure 1).

Additional plant-derived enzymes such as the delta-7-reductase gene can be incorporated to enhance biosynthetic efficiency, alongside other regulatory genes like StAR (Steroidogenic Acute Regulatory Protein) for improved cholesterol transport, and HSD17B (17β-Hydroxysteroid Dehydrogenase) to convert estrone into the more potent estradiol (Table 1). Introducing such supportive enzymes ensures that the biosynthetic pathway operates efficiently, maximizing hormone yield and fidelity to human steroidogenesis [26,27].

This way, human-identical hormones can be manufactured with improved environmental and process safety, lower energy requirements, reduced generation of toxic byproducts, and fewer harsh chemical processes. This is because conventional steroid hormone manufacturing depends on multi-step chemical transformations of plant-derived sterols using organic solvents, strong acids, oxidizing agents, and high-energy conditions, which generate hazardous waste and contribute to environmental burden. Furthermore, the proposed method offers advantages in sustainability and scalability. In addition, microbial fermentation platforms are inherently scalable, allowing efficient large-scale manufacturing using established industrial bioprocessing systems [27,28,29].

### 2.2. AI-Controlled Pulsatile Hormone Delivery

**Figure 2:**
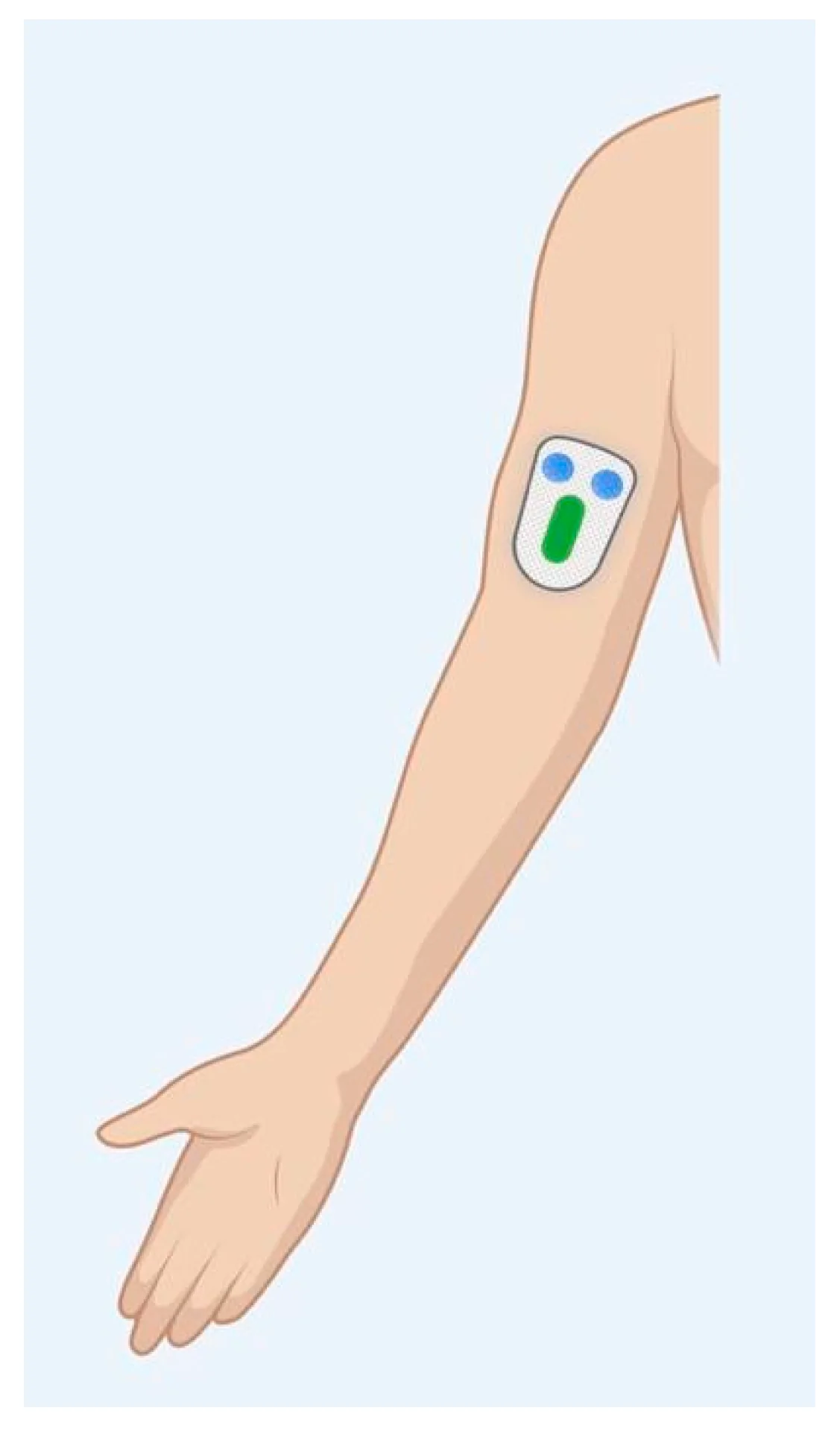
A subcutaneous pulsatile pump. An illustration of the programmed AI dosing system to release precise hormone doses that simulate the hormonal fluctuations of the menstrual cycle. Designed by Biorender.com.

Rather than administering HRT in conventional forms, hormones are delivered via a subcutaneous pulsatile pump, programmed to release micro-doses that simulate the hormonal fluctuations of the menstrual cycle (Figure 2).

**Figure 3:**
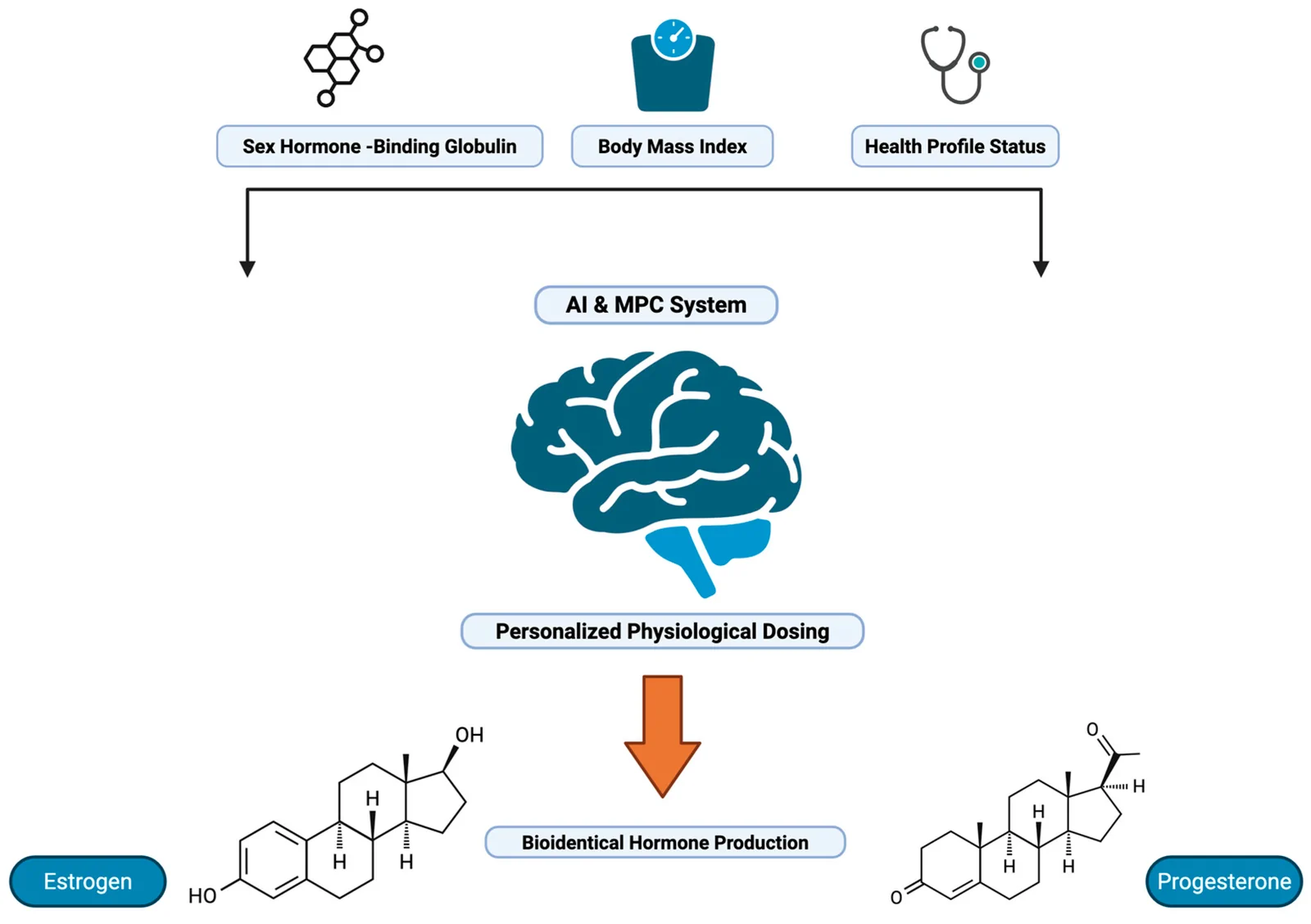
An AI-guided system for personalized and physiological bioidentical hormone dosing. This diagram illustrates a semi-closed-loop system for generating personalized and physiologically relevant dosing for bioidentical hormone production using an AI dosing system. Designed by Biorender.com.

An integrated AI module adjusts hormone levels according to individual patient factors such as weight, health status, and metabolic profile, ensuring personalized and physiologic dosing [30,31,32] (Figure 3).

We take into account that ovarian hormone secretion is under the influence of the pituitary gland, which releases follicle stimulating hormone (FSH) and luteinizing hormone (LH) in a pulsatile manner (approximately one pulse every 60–90 min during the follicular phase, and one pulse every 2 to 3 h during the luteal phase), but in return, the ovaries do not immediately secrete 17β-estradiol and progesterone in direct response to each pulse. Instead, there is a range of hormonal concentrations that correspond to each phase of the menstrual cycle (Table 2), influenced by both internal and external factors [12].

The proposed system enables dynamic, time-varying hormone delivery within physiologic ranges, allowing coordinated modulation of 17β-estradiol and progesterone levels over time. Rather than maintaining fixed hormone concentrations, the system adjusts the relative proportions of both hormones in a controlled manner, approximating physiologic patterns of hormonal variation while remaining within clinically defined safety ranges. Moreover, by integrating artificial intelligence (AI) to select the optimal hormone dose from this range appropriate for each woman based on periodically analyzing individual data, the system thereby achieves personalized, adaptive endocrine regulation. The system is configured to specific parameters to illustrate the primary workflow.

**Figure 4:**
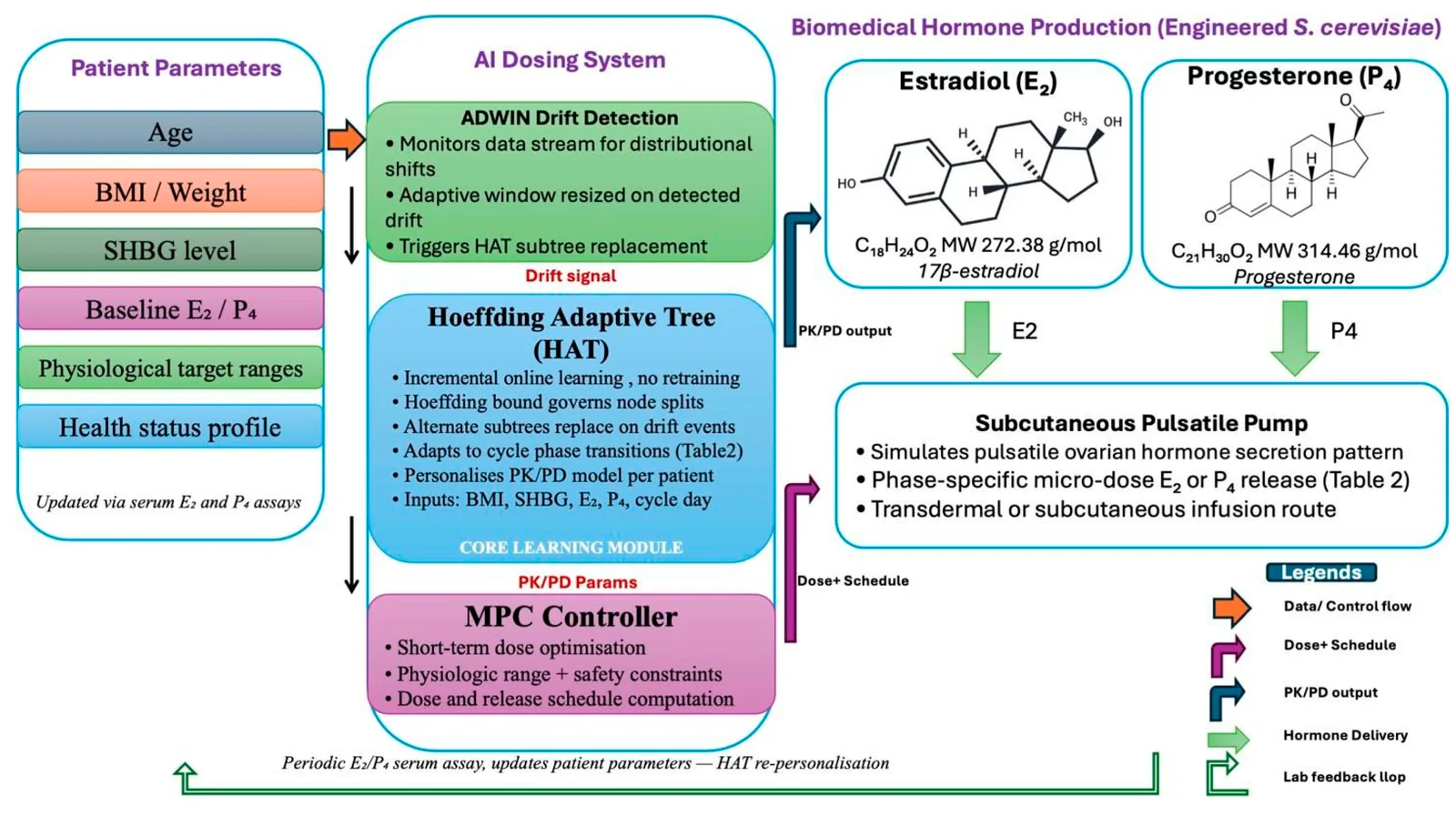
The workflow of the AI-guided personalized bioidentical hormone dosing system incorporating the Hoeffding Adaptive Tree (HAT).

Patient-specific parameters including, age, BMI, sex hormone-binding globulin (SHBG), baseline 17β-estradiol (E2) and progesterone (P4), menstrual cycle phase/day, and health status profile, are streamed into the AI dosing system (purple dashed container). The ADWIN Drift Detector monitors the incoming data stream for distributional shifts arising from physiological changes (weight fluctuation, variation in hormones level, transitions, SHBG variation) and signals the need for model adaptation. The Hoeffding Adaptive Tree (HAT) performs incremental online learning without full model retraining: the Hoeffding bound determines statistically sufficient conditions for node splits, while ADWIN-triggered alternate subtrees replace outdated branches, ensuring the pharmacokinetic/pharmacodynamic (PK/PD) model remains calibrated to the patient’s current physiology. Updated PK/PD parameters are forwarded to the model predictive control (MPC) Controller, which computes the optimal short-term dose and release schedule within predefined physiologic safety ranges. Bioidentical 17β-estradiol (C_1_H_24_O_2_; MW 272.38 g/mol) and progesterone (C_21_H_30_O_2_; MW 314.46 g/mol), biosynthesized via engineered *Saccharomyces cerevisiae*, are delivered through a subcutaneous pulsatile pump that maintains phase-specific hormone concentrations of the natural menstrual cycle (Table 2). The semi-closed feedback loop (green dashed, Figure 4) is updated via periodic bi-weekly serum E2/P4 assays, refreshing patient parameters and prompting HAT re-personalization. All flow directions are indicated by arrowheads; the dashed lines denote optional or feedback pathways. BMI, body mass index; MPC, model predictive control; PK/PD, pharmacokinetic/pharmacodynamic; and SHBG.

#### Adaptive Dosing Personalization via Hoeffding Adaptive Tree

The proposed AI module employs a Hoeffding Adaptive Tree (HAT) as its core learning algorithm, selected for its ability to perform continuous, incremental learning from periodically updated patient data without requiring full model retraining [30]. Unlike batch-learning models, HAT uses the Hoeffding bound, a statistical guarantee, to determine when a decision node has received sufficient data to justify a split, enabling reliable tree growth from limited observations [30].

A critical advantage of HAT in this clinical context is its built-in concept drift detection via the ADWIN (Adaptive Windowing) mechanism. Hormonal pharmacokinetics and patient physiology are inherently non-stationary: body weight fluctuates, SHBG levels shift seasonally, and the patient’s menstrual cycle phase transitions between the follicular, preovulatory, and luteal stages, each with distinct target hormone ranges (Table 2). When ADWIN detects a statistically significant change in the incoming data stream, HAT automatically replaces outdated subtrees with newly trained alternates, ensuring the model remains calibrated to the patient’s current physiological state without clinical intervention [33].

The model receives the following input features: body mass index (BMI), SHBG con-centration, baseline estradiol (E2) and progesterone (P4) levels, and patient age. Its output consists of updated pharmacokinetic (PK) parameters, specifically hormone half-life estimates and distribution volumes, which are passed to the model predictive control (MPC) layer to compute the optimal pulsatile dose and release schedule for the subcutaneous pump (Figure 3).

Because no clinically validated real-time steroid hormone sensor is currently available, the HAT model operates on a semi-closed-loop basis: it adapts its internal PK model continuously using routine clinical measurements (e.g., bi-weekly serum E2 and P4 assays), rather than millisecond-level sensor feedback as in closed-loop insulin delivery systems [34]. It is important to clarify that the bi-weekly measurement interval and the system’s short-term pulsatile dosing operate on fundamentally different time scales by design, rather than representing a control-loop inconsistency. Short-term dosing decisions are generated entirely by the MPC layer using pharmacokinetic and pharmacodynamic modeling of hormone half-life, clearance, and distribution, the same modeling approach used regardless of when the last measurement occurred. The bi-weekly serum assays serve a distinct, slower time scale function: recalibrating the underlying PK/PD model’s patient-specific parameters as physiological conditions drift, via the HAT/ADWIN mechanism described above. In this architecture, safety during the interval between measurements is maintained by the MPC layer operating within predefined physiologic safety ranges (Table 2) derived from population pharmacokinetic data, with the system defaulting to conservative, previously validated dosing parameters if drift detection flags a discrepancy requiring clinical reassessment rather than autonomous adjustment beyond the safety envelope. This architecture allows short-term dose optimization through MPC and long-term physiological adaptation through HAT, together maintaining hormone concentrations within predefined safety ranges across all cycle phases.

## 3. Evaluation of the Hypothesis

The proposed system introduces a novel, multi-axis strategy to overcome the key shortcomings of current hormone replacement therapy (HRT).

### 3.1. Clinical and Scientific Value

By combining human-identical hormone production, physiologic pulsatile delivery, and AI-driven personalization technology, the proposed system directly addresses the chemical and pharmacologic limitations of current HRT. These include binding to non-target receptors due to structural differences between synthetic and endogenous hormones, industrial challenges inherent to semi-synthetic manufacturing processes, first pass hepatic metabolism in the oral administration, continuous hormone release such as transdermal administration, and the loss of the natural cyclic and dynamic pattern of ovarian hormone secretion. While the molecular identity of the final hormone product is independent of its production route, purity and batch-to-batch consistency are not. The multi-step chemical conversion required to finish plant-sterol-derived bioidentical hormones introduces greater opportunity for process-related impurities and inter-batch variability than a single-pathway enzymatic biosynthesis route. This distinction carries particular clinical relevance for the proposed system, which relies on continuous, automated micro-dosing rather than infrequent standard-dose administration: at this level of dosing precision, hormone purity and consistency directly affect dosing accuracy and safety margins in a way that is less consequential for conventional HRT formulations. This integrated approach is expected to mirror the natural secretion patterns of 17β-estradiol and progesterone, closely reproducing the hormonal fluctuations of the menstrual cycle, and enable personalized therapy, with AI algorithms adjusting dosing to each woman according to weight, metabolic profile, and SHBG, improving tolerability and long-term adherence. Collectively, these advantages have the potential to restore the natural effects of 17β-estradiol and progesterone, advance precision endocrine therapy, and may inform future therapies.

### 3.2. Feasibility

Although the proposed system presents significant challenges, it remains scientifically achievable because it integrates several well-established technologies, with targeted updates to meet the system’s specific goals. At the biotechnological level, yeast such as *Saccharomyces cerevisiae* are already used to produce complex human proteins (e.g., recombinant insulin) by transferring human genes [29]. The proposed approach involves engineering yeast with key human genes to synthesize progesterone and 17β-estradiol to achieve hormones that are identical to natural human steroids [28]. On the delivery side, there are closed loop systems that are already mature, such as an artificial pancreas for diabetes management [35]. These systems typically consist of four main components: (1) a continuous glucose monitor (CGM) that measures interstitial glucose levels in near real time, (2) a control algorithm that processes sensor data and determines the required insulin dose, (3) a programmable insulin pump that delivers insulin subcutaneously, and (4) a closed feedback loop that updates dosing decisions based on incoming glucose measurements. But the proposed system operates as a semi-closed loop due to the lack of clinically available real-time steroid hormone sensors as in an artificial pancreas, which relies on blood sugar sensors to deliver precise amounts of insulin. The proposed system delivers hormones through the combination of (1) a programmable drug-delivery device and (2) a computational dosing engine designed to maintain physiologic concentrations of 17β-estradiol and progesterone within predefined safety ranges. The proposed device comprises: (1) a reservoir containing purified 17β-estradiol and progesterone formulations, (2) a miniaturized pump or controlled release module, and (3) a clinician-facing dashboard for target setting and safety oversight. The delivery system could be implemented as a transdermal or subcutaneous microinfusion pump [35,36].

A reasonable concern with the proposed delivery architecture is that a subcutaneous pump-based system introduces a device-management burden not present with oral therapy, which could reduce treatment adherence relative to a daily tablet. This trade-off is not unique to the proposed system. It has been studied extensively in insulin pump therapy for type 1 diabetes, offering a relevant precedent. Despite requiring more active device management than injections, pump therapy is associated with lower all-cause mortality and lower rates of diabetic ketoacidosis compared with multiple daily injections, based on large real-world cohort data [37]. Pump adoption also remains substantial in health systems with adequate support infrastructure, reaching approximately 40% among individuals with type 1 diabetes in the United States. However, this benefit is not unconditional. A UK randomized controlled trial found that pumps conferred no clear cost-effectiveness advantage when both pump and injection groups received equivalent structured patient education [38]. This suggests that the burden of device-based therapy is mitigated by adequate training and support, rather than eliminated by the device itself. The same logic applies to the proposed hormone-delivery pump: its adherence burden is real, but not inherently prohibitive, provided the system includes appropriate patient education and support. The semi-closed-loop AI architecture also reduces ongoing patient decision burden specifically. Routine dosing adjustments occur automatically between bi-weekly clinical assessments, rather than requiring continuous manual titration as in earlier-generation pump technologies. Still, adherence to this specific delivery modality has not been directly studied in the ovarian hormone context. It would need to be evaluated prospectively as part of the system’s staged validation pathway.

The model predictive control (MPC) system within the proposed semi-closed-loop system is designed to be model-based rather than sensor-based, reflecting the current absence of clinically available real-time steroid hormone sensors. Instead of relying on continuous biochemical measurements, the MPC utilizes pharmacokinetic and pharmacodynamic models of 17β-estradiol and progesterone to estimate hormone levels following administration. At the short time scale, the MPC calculates the optimal dose and release schedule based on predicted hormone levels, incorporating parameters such as hormone half-life, metabolic clearance, and distribution characteristics. This enables controlled delivery that minimizes deviations from predefined physiologic target ranges while respecting safety constraints. At a longer time scale (weeks to months), an adaptive AI module personalizes the model by updating patient-specific parameters using clinical inputs, including body mass index (BMI), SHBG, and baseline E2/P4. Rather than directly determining doses, the AI module adjusts the underlying pharmacokinetic model, thereby refining the MPC’s future predictions and recommendations in accordance with patient-specific variation. Together, this architecture enables short-term dose optimization through MPC and long-term individualized adaptation through AI, aiming to maintain hormone concentrations within clinically appropriate physiologic ranges [30,31,32,33,39].

The implementation pathway would proceed in defined stages: (1) Human-identical hormone production using engineered yeast biotransformation. (2) Device development, ensuring chemical stability of the hormones within the pump and protection from external influences. (3) Integration of the programmable delivery device with the model-based control framework would be undertaken, including validation of dosing accuracy and safety constraints. A more detailed development and validation pathway, encompassing hormone purification, formulation stability, device computability, pharmacokinetic modeling, and staged preclinical and clinical evaluation, is outlined in Table 3.

Importantly, each core component of the system, microbial steroid biosynthesis, pharmaceutical purification of human-identical hormones, programmable subcutaneous or transdermal pump technology, and model predictive control algorithms, has been independently validated in medical or industrial contexts. Although adaptive AI-assisted personalization remains an emerging area, its application in medicine and medical device design is expanding rapidly. Therefore, integrating these established technologies into a unified therapeutic platform is ambitious but technically feasible, provided that systematic engineering optimization, preclinical evaluation, and staged clinical trials are conducted [40,41].

## 4. Hypothesis Testing

The goal of the proposed AI ovarian system is to restore the natural physiological effects of ovarian hormones, effects that current hormone replacement therapy (HRT) has significant difficulty reproducing due to limitations related to the oral route of administration, the chemical form of synthetic hormones, and the non-physiologic single dose delivery pattern. By combining bioengineered hormone production, artificial intelligence, and pulsatile pump technology, the system seeks to mimic natural ovarian secretion both chemically and dynamically.

The rationale for pursuing bioidentical rather than synthetic hormones is well-informed in the existing literature. Conjugated equine estrogens and synthetic progestogens, which have historically dominated HRT formulations, differ substantially in their molecular structure and receptor binding profiles from endogenous ovarian hormones [25,42]. These structural differences translate into clinically meaningful divergences in downstream effects. For instance, synthetic progestogens such as medroxyprogesterone acetate interact with androgen, glucocorticoid, and mineralocorticoid receptors in ways that natural progesterone does not, producing metabolic and cardiovascular side effects that are not attributable to the hormone class as a whole but rather to the specific molecule used [24]. This distinction is not merely pharmacological but has real consequences for patient safety, as the Women’s Health Initiative trial, which used conjugated equine estrogens combined with medroxyprogesterone acetate, reported elevated risks of breast cancer, venous thromboembolism, and cardiovascular events that have not been consistently replicated with bioidentical formulations [18,20]. The use of bioengineered estradiol and progesterone produced through yeast-based biosynthetic pathways, as proposed in this system, therefore represents a scientifically justified departure from conventional synthetic preparations.

On the biotechnology side, the feasibility of microbial hormone biosynthesis is now well-established. Steroid biosynthesis in engineered *Saccharomyces cerevisiae* has been demonstrated for hydrocortisone, pregnenolone, progesterone, and cholesterol derivatives, confirming that the yeast cellular machinery can be redirected to support complex steroidogenic pathways with relatively modest genetic modifications [27,28]. More recent work has refined these approaches further, with strategies involving organelle-level engineering and pathway reconstruction yielding increasingly efficient sterol and steroid outputs [26,43]. The parallel success of recombinant insulin production in yeast, now the dominant industrial method for insulin manufacturing, demonstrates that the translation of such biosynthetic strategies from laboratory to clinical-grade production is not merely theoretical [29,44]. The AI ovarian system builds on this established biotechnological foundation and extends it toward a new therapeutic application.

What most fundamentally distinguishes this proposal from existing HRT approaches, however, is not the chemistry of the hormones but the delivery architecture. Natural ovarian function is characterized by pulsatile secretion patterns that are tightly regulated by hypothalamic gonadotropin-releasing hormone (GnRH) pulses and responsive to systemic feedback signals [45]. While pulsatile secretion of GnRH and gonadotropins is well-established, direct evidence also demonstrates that ovarian steroid output itself is pulsatile rather than smoothly continuous. Frequent serial sampling studies have detected episodic fluctuations in circulating estradiol and progesterone concentrations across the menstrual cycle [46], and the corpus luteum has been shown to co-secrete estradiol and progesterone in discrete pulses that are temporally linked to LH pulses [47], indicating that short-interval ovarian steroid pulsatility is not merely inferred from upstream GnRH dynamics but is directly observable and mechanistically coupled to it. This pulsatility is not incidental, it is physiologically essential. The amplitude and frequency of hormone pulses govern receptor sensitivity, downstream gene expression, and the integration of hormonal signals across multiple target tissues [48]. Conventional HRT, whether oral or transdermal, delivers hormones in a sustained, non-pulsatile manner that bears little resemblance to this dynamic pattern. Transdermal delivery does offer meaningful advantages over oral administration in terms of avoiding first pass hepatic metabolism and reducing thrombotic risk, as oral 17β-estradiol has been shown to increase clotting factors and venous thromboembolism risk through hepatic activation in ways that transdermal preparations largely do not [16,17]. Nevertheless, even transdermal HRT provides static rather than pulsatile hormone exposure, and this physiological gap is what the AI-driven pump component of the proposed system directly addresses.

The precedent for closed-loop, AI-regulated endocrine replacement is already established in diabetology. The artificial pancreas, a system combining continuous glucose monitoring, predictive algorithms, and automated insulin delivery, has demonstrated in multiple clinical trials that real-time feedback-controlled hormone delivery can replicate the responsiveness of biological endocrine systems more realistically than fixed dose regimens [31,32,34,49]. The control strategies employed in these systems, including model predictive control and adaptive algorithms, have proven capable of managing the rapid glucose fluctuations that accompany exercise, meals, and sleep in a way that rigid dosing schedules cannot [31,39,49]. Translating this logic to ovarian hormone replacement is conceptually coherent: just as the artificial pancreas responds to deviations in blood glucose by modulating insulin secretion, the AI ovarian system would respond to measured circulating hormone levels by modulating 17β-estradiol and progesterone delivery. The greater complexity of the ovarian hormone system, involving multiple hormones, circadian variation, and longer-cycle dynamics, represents an engineering challenge but not a fundamental conceptual barrier, particularly given the rapid advances in AI-based physiological modeling and medical device integration [40,50]. The physiological consequences of restoring pulsatile 17β-estradiol and progesterone delivery are potentially far-reaching, given the breadth of tissues that express 17β-estradiol and progesterone receptors. Cardiovascular protection may also represent a clinically meaningful benefit of this approach. 17β-estradiol has well-characterized endothelial effects, promoting nitric oxide production, reducing oxidative stress, and modulating lipid profiles in ways that are associated with reduced atherosclerotic risk [6,8]. Clinical data support a timing hypothesis in which the cardiovascular benefit of 17β-estradiol is most pronounced when therapy is initiated close to the menopausal transition rather than years after it, consistent with a window of opportunity during which endothelial function remains responsive [19,51]. The DANISH trial and related studies have shown that early initiation of HRT in recently menopausal women reduces cardiovascular event rates, while delayed initiation may not confer the same benefit and could be harmful in women with established atherosclerosis [51]. Hot flushes themselves have been linked to adverse vascular indices including elevated aortic calcification and carotid intima media thickness, suggesting that inadequately treated menopausal transition carries its own vascular cost [52]. By restoring a hormone environment that more closely resembles premenopausal physiology, the AI ovarian system may plausibly mitigate these risks more effectively than conventional HRT.

Bone protection represents another critical outcome domain. The decline in 17β-estradiol at menopause accelerates osteoclast activity and leads to progressive bone mineral density loss, contributing substantially to postmenopausal fracture risk [10]. Progesterone, independently of 17β-estradiol, has direct anabolic effects on bone through osteoblast stimulation, and combined 17β-estradiol-progesterone therapy has been shown to produce greater gains in spinal bone mineral density than estrogen alone [11]. The genitourinary and pelvic floor consequences of 17β-estradiol deficiency, including vaginal atrophy, urinary symptoms, and pelvic floor laxity, also respond to hormonal restoration, and these too may plausibly benefit from a system delivering physiologically representative hormone levels [12,53].

The neuroprotective dimension of this system deserves particular attention. 17β-estradiol receptors are expressed throughout the brain in regions governing cognition, mood, and neuroplasticity, and 17β-estradiol has been shown to support synaptic density, cholinergic transmission, and mitochondrial function in neural tissue [54,55]. Progesterone similarly exerts neuroprotective effects through both classical receptor pathways and through its conversion to neuroactive steroids such as allopregnanolone, which modulates GABAergic activity and has been demonstrated to reduce neuroinflammation and support brain injury recovery [3,56]. The loss of these neuroprotective hormonal influences at menopause may contribute to the cognitive changes and mood disturbances frequently reported during the menopausal transition, and there is growing interest in whether earlier and more physiologically accurate hormone restoration could reduce dementia risk. These possibilities strengthen the case for a system capable of delivering hormones in a pattern that more closely mirrors endogenous secretion rather than providing the blunt pharmacological exposures associated with conventional replacement.

The AI infrastructure of the proposed system is not a peripheral feature but a central functional component. Machine learning models trained on biosensor data streams could identify individual hormonal patterns, predict endogenous secretory cycles, and adjust delivery parameters accordingly, functions that have direct parallels in the personalized dosing algorithms already deployed in advanced diabetes management systems [30,31,32]. AI has increasingly been incorporated into FDA-authorized medical devices, though regulatory frameworks specifically addressing continuously adaptive, closed-loop clinical algorithms, as would be required for this system, remain an active area of development [50]. Furthermore, advances in AI-based diagnostics and biomarker interpretation have demonstrated that machine learning systems can identify clinically meaningful patterns in hormone data with a sensitivity that exceeds conventional threshold-based interpretation [41,57], suggesting that the AI component of the proposed system could add diagnostic as well as therapeutic value over time.

The hypothesis is testable through several phases. Initially, in vitro assays could confirm the biochemical equivalence of bioengineered 17β-estradiol and progesterone to endogenous hormones. Subsequent preclinical models could evaluate vascular and metabolic responses as well as neurotransmitter regulation, followed by clinical trials assessing lipid modulation, endothelial function, bone mineral density using DEXA scanning, and symptoms related to ovarian failure. Progesterone’s known influence on insulin sensitivity and glucose metabolism [9] would also need to be accounted for in the metabolic safety profiling of the system, particularly given the risk of unintentionally inducing insulin resistance through non-physiological progesterone delivery patterns.

Regarding long-term safety, it is important to acknowledge that concerns about endometrial and breast cancer risk with combined HRT cannot be dismissed, even when bioidentical hormones are used, since the mitogenic effects of 17β-estradiol on reproductive tissue are hormone-class effects rather than synthetic artifacts [20,21]. However, the evidence suggests that the risk profile of bioidentical progesterone differs meaningfully from that of synthetic progestogens, particularly with respect to breast tissue proliferation [24], and a system that delivers progesterone in appropriately timed pulses rather than continuously may further reduce exposure-related risks. The question of what constitutes optimal pulsatile progesterone delivery to protect the endometrium while minimizing systemic side effects is one that this system’s AI component would be uniquely positioned to address through adaptive titration.

If validated, the AI ovarian system has the potential to meaningfully contribute to the evolution of menopausal therapy, transforming static hormone replacement into a dynamic, self-regulating endocrine platform. Furthermore, this success could open the door for similar biotechnological and AI-integrated approaches in other endocrine disorders, including therapies involving adrenal or thyroid hormones. The integration of synthetic biology, semi-closed-loop control engineering, and clinical AI reflects an emerging trajectory in biomedical technology [40,58], and the ovarian hormone system, with its well-characterized physiology, measurable biosensor endpoints, and large patient population, may prove to be a potential early application of this integrated approach.

## 5. Conclusions

Women with ovarian insufficiency due to primary or secondary causes suffer from multiple symptoms and comorbidities due to the loss of ovarian hormone function. Current HRT strategies have limitations in fully restoring these physiological benefits, highlighting the need for a more precise and physiologic approach.

The proposed system addresses these shortcomings by producing human-identical hormones through engineered yeast, which are designed to achieve selective binding to the same receptors as endogenous hormones. This approach offers additional advantages, including a lower environmental impact, the use of renewable feedstocks, and high scalability. Physiologic secretion is maintained via an AI-guided, pulsatile transdermal/subcutaneous pump that replicates the natural timing and dosing patterns of ovarian hormone release. In this way, the system aims to restore the physiological effects of 17β-estradiol and progesterone while minimizing side effects and supporting more personalized, long-term health outcomes compared to conventional HRT.

## Figures and Tables

**Table 1:** Genetic modifications and functional components of engineered *Saccharomyces cerevisiae* used for biosynthesis of human steroid hormones [28].

Component	Function/Action	Example Genes
Simple Carbon Sources	Simple carbon sources	Ethanol, Galactose
Host Organism	*Saccharomyces cerevisiae*	*Saccharomyces cerevisiae*
Transferred Genes	Essential for converting precursors to final hormones	3β-HSD, *CYP17A1*, *CYP21A1*, *CYP11A1*, *HSD3B1*, *CYP19A1*, cytochrome P450 SCC, adrenodoxin reductase
Enhancing Genes	Improve biosynthetic efficiency and final product potency	delta-7-reductase
	Regulate cholesterol supply	StAR (Steroidogenic Acute Regulatory Protein)
	Convert estrone to more potent estradiol	HSD17B (17β-Hydroxysteroid Dehydrogenase)
Modified/Deleted Genes	Eliminate unwanted side reactions, particularly during pregnenolone formation	*ERG5*, *ATF2*, *GCY1*, *YPR1*
Target Products	Final desired steroid hormones	Estrogen (e.g., estradiol), Progesterone

**Table 2:** The daily production rate of hormones during different stages of the menstrual cycle [2].

Hormones	Early Follicular	Preovulatory	Midluteal
Progesterone (mg)	1	4	25
17a-Hydroxyprogesterone (mg)	0.5	4	4
Estrone (ug)	50	350	250
Estradiol (ug)	36	380	250

**Table 3:** Proposed development and validation pathway.

Stage	Focus	Key Activities
1. Hormone production & purification	Biosynthesis and purity	Yeast biotransformation; chromatographic purification; HPLC/MS characterization for identity and impurity profiling
2. Formulation & stability	Chemical stability	Thermal stability, degradation kinetics, reservoir/delivery-line material compatibility over intended use period
3. Pump & device engineering	Delivery hardware	Dosing accuracy validation, reservoir integrity, resistance to temperature/mechanical stress; builds on diabetes pump platforms
4. Pharmacokinetic modeling	PK/PD foundation	Population and individual PK/PD models for half-life, clearance, distribution; basis for MPC dosing layer
5. Preclinical evaluation	Biological validation	In vitro/animal models confirming biochemical equivalence, vascular/metabolic response, physiologic pulsatile profile fidelity
6. Staged clinical evaluation	Safety and efficacy	Small-cohort safety/dosing accuracy studies → larger trials on lipid modulation, endothelial function, BMD, symptom resolution; predefined safety stopping rules at each stage

## Data Availability

No new data were created or analyzed in this study. Data sharing is not applicable to this article.
